# MAP3K8 Is a Prognostic Biomarker and Correlated With Immune Response in Glioma

**DOI:** 10.3389/fmolb.2021.779290

**Published:** 2021-12-23

**Authors:** Jing Ren, Yixin Xu, Jia Liu, Sicheng Wu, Ruihan Zhang, Haowei Cao, Jinmin Sun

**Affiliations:** ^1^ Jiangsu Key Laboratory of Brain Disease Bioinformation, Research Center for Biochemistry and Molecular Biology, Xuzhou Medical University, Xuzhou, China; ^2^ Department of General Surgery, The Affiliated Hospital of Xuzhou Medical University, Xuzhou, China; ^3^ Institute of Digestive Diseases, Xuzhou Medical University, Xuzhou, China; ^4^ Department of Pathology, The Affiliated Hospital of Xuzhou Medical University, Xuzhou, China; ^5^ Laboratory of Clinical and Experimental Pathology, Department of Pathology, Xuzhou Medical University, Xuzhou, China

**Keywords:** MAP3K8, prognostic biomarker, glioma, macrophage, immune infiltration, immunomodulators

## Abstract

MAP3K8 is a serine/threonine kinase that is widely expressed in immune cells, non-immune cells, and many tumor types. The expression, clinical significance, biological role, and the underlying molecular mechanisms of MAP3K8 in glioma have not been investigated yet. Here, we discovered that MAP3K8 was aberrantly overexpressed in glioma and correlated with poor clinicopathological features of glioma by analysis on different datasets and immunohistochemistry staining. MAP3K8 is an independent prognostic indicator and significantly correlates with the progression of glioma. We also performed the function and pathway enrichment analysis of MAP3K8 in glioma to explore its biological functions and underlying molecular mechanisms in glioma. MAP3K8 co-expressed genes were mainly enriched in immune-related biological processes such as neutrophil activation, leukocyte migration, neutrophil-mediated immunity, lymphocyte-mediated immunity, T-cell activation, leukocyte cell–cell adhesion, regulation of leukocyte cell–cell adhesion, B-cell-mediated immunity, myeloid cell differentiation, and regulation of cell–cell adhesion. Single-cell RNA sequencing data and immunohistochemistry analysis demonstrated that MAP3K8 is expressed in malignant and immune cells and mainly enriched in the microglia/macrophage cells of glioma. The expression of MAP3K8 was positively correlated with immune infiltration, including effector memory CD4^+^ T cells, plasmacytoid dendritic cells, neutrophils, myeloid dendritic cells, mast cells, and macrophage in glioma. Further correlation analysis demonstrated that a series of inhibitory immune checkpoint molecules, chemokines, and chemokine receptors was positively correlated with the expression of MAP3K8. MAP3K8 might play an essential role in tumor immunity, and inhibition of MPA3K8 is a plausible strategy for glioma immunotherapy.

## Introduction

Glioma is the most prevalent and lethal primary brain tumor that typically arises from glial or precursor cells (Stupp et al., 2005; Lapointe et al., 2018; Ostrom et al., 2020). Conventional therapies such as maximal surgical resection, chemotherapy of temozolomide, and radiotherapy are considered to be the best available therapeutic approaches (Gusyatiner and Hegi, 2018; Frosina, 2021). Currently, immunotherapy has been used as a promising strategy with the ability to penetrate the blood–brain barrier. However, limited improvements in the prognosis of glioma patients have been achieved, and only a few drugs have been approved for the treatment of glioma (Norden and Wen, 2006; Korber et al., 2019). To date, there is a lack of effective treatment regimen for patients with glioma. Therefore, it is urgent to disclose the cellular and molecular mechanisms of glioma development and discover promising molecular markers for diagnosis, prognosis, and potential therapeutic target.

MAP3K8 is a serine/threonine kinase that is widely expressed in immune and non-immune cells and originally identified as a proto-oncogene and a target for provirus integration (Patriotis et al., 1993; Erny et al., 1996; Xu et al., 2018). Over the years, MAP3K8 has been identified as a molecular linchpin that connects inflammation, tumorigenesis, and tumor immunity (Njunge et al., 2020). MAP3K8 was involved in the inflammatory and immune cell recruitment, differentiation, and activation by regulating the production of pro-inflammatory cytokines, chemokines, enzymes, and growth factors (Gantke et al., 2012; Lee et al., 2015; Njunge et al., 2020). Overexpression of MAP3K8 was found in several tumor types, including skin cancer, prostate cancer, breast cancer, squamous cell carcinoma, ovarian cancer, hepatocellular carcinoma, and colorectal cancer (Sourvinos et al., 1999; Jeong et al., 2011; Gruosso et al., 2015; Lee et al., 2015; Li et al., 2015; Pyo et al., 2018). The enhanced expression and activity of MAP3K8 were associated with poor prognosis and implicated in tumor progression such as cell proliferation, migration, invasion, stemness, angiogenesis, epithelial-to-mesenchymal transition, and metastasis (Vougioukalaki et al., 2011; Gantke et al., 2012; Gruosso et al., 2015). The overexpression of MAP3K8 in murine salivary glands induced tumorigenesis of squamous cell carcinoma (Lee et al., 2019). In thyroid cancer stem cells, MAP3K8 was involved in resistance to vemurafenib and represented a potential novel prognostic marker and therapeutic target (Giani et al., 2019). In ovarian carcinomas, MAP3K8 promoted cell proliferation and migration by regulating the cell cycle and adhesion dynamics. Nevertheless, the expression, clinical significance, and the biological role of MAP3K8 in glioma have yet to be investigated and remain unclear. Especially, the role of MAP3K8 in glioma immune cells and its underlying molecular mechanisms are still unclear.

Here, we found that MAP3K8 was aberrantly overexpressed in glioma and correlated with poor clinicopathological features. MAP3K8 is an independent prognostic indicator and significantly correlates with the disease progression of glioma. We also performed function and pathway enrichment analysis to explore the biological function and underlying molecular mechanisms of MAP3K8 in glioma. Single-cell RNA sequencing (RNA-seq) data and immunohistochemistry (IHC) analysis showed that MAP3K8 was expressed in malignant and immune cells and mainly enriched in the microglia/macrophage cells of glioma. Biological processes such as neutrophil activation, leukocyte migration, neutrophil-mediated immunity, lymphocyte-mediated immunity, and T-cell activation were enriched. Correlation analysis of MAP3K8 with immune infiltration in glioma was performed. Meanwhile, MAP3K8 was positively correlated with several immune checkpoint molecules, chemokines, and chemokine receptors. MAP3K8 might play an essential role in tumor immunoregulation, which leads to pro-tumorigenic inflammation. In a nutshell, MAP3K8 is a prognostic biomarker and is correlated with immune response in glioma.

## Materials and Methods

### Human Tissue

Glioma tissues (*n* = 94) were obtained from the Department of Pathology of the Affiliated Hospital of Xuzhou Medical University between 2016 and 2017. All specimens were identified by pathologists according to the 2016 World Health Organization (WHO) classification criteria. Ethical approval for this study was obtained from the Institutional Ethics Committee of Affiliated Hospital of Xuzhou Medical University (no. XYFY2018-KL056-01).

### Data Acquisition

RNA-seq data were downloaded from The Cancer Genome Atlas (TCGA) database (*n* = 703), the Chinese Glioma Genome Atlas (CGGA) database (batch I, *n* = 413; batch II, *n* = 273), and Genotype-Tissue Expression (GTEx, *n* = 1,157). For overall survival and expression analysis, 413 glioma tissues of dataset batch I and 273 glioma tissues of dataset batch II from the CGGA were used after the deletion of incomplete data [grade, overall survival, isocitrate dehydrogenase (IDH) mutation status, 1p/19q codeletion status, and *O* ^6^-methylguanine-DNA methyltransferase promoter methylation status]. For the gene expression profiles across all tumor samples and paired normal tissues, the Gene Expression Profiling Interactive Analysis web server (http://gepia.cancer-pku.cn/detail.php) was used.

### Cell Culture

Human glioblastoma multiforme (GBM) cell lines (U87, U118, U251, LN229 and T98G), human normal brain glial cells (HEB), normal human astrocyte (NHA), and primary glioma cell lines were cultured in Dulbecco’s modified Eagle’s medium (DMEM; Keygen Biotech, Nanjing, China) supplemented with 10% fetal bovine serum (FBS; Life Technologies, Carlsbad, CA, USA). The Committee of Type Culture Collection of Chinese Academy of Sciences (Shanghai, China) provided short tandem repeat DNA fingerprinting for GBM cell line identity confirmation in August 2018.

### Immunohistochemistry Analysis

The brain tissues of mice were fixed in 4% paraformaldehyde and embedded in paraffin. IHC was performed as per standard laboratory protocols as previously described (Yu et al., 2019). In brief, paraffin blocks were sliced into 4-μm sections and the sections were deparaffinized and rehydrated *via* a graded series of ethanol. Antigen retrieval was autoclaved in sodium citrate buffer with high pressure for 3 min. Then, the sections were treated with 3% hydrogen peroxide and blocked with 10% goat serum. Subsequently, the sections were incubated with anti-MAP3K8 antibody (1:500 dilution; Santa Cruz, CA, United States) overnight. Thereafter, the sections were incubated with horseradish peroxidase-labeled secondary antibodies for 1 h and DAB for 2–5 min at room temperature, followed by hematoxylin staining. Photomicrographs were acquired using an Olympus microscope and scored by two pathologists using a double-blind method.

### Western Blot

The collected cells were lysed in RIPA buffer, and the protein concentration was detected using the BCA Protein Assay Kit (Beyotime, Shanghai, China). The lysates were separated with SDS-PAGE gel and transferred onto nitrocellulose membranes (Millipore, Burlington, MA, United States). The primary antibodies for MAP3K8 (1:500 dilution; Santa Cruz, CA, United States) and GAPDH (1:10,000; ProteinTech, Rosemont, IL, United States) were used. Membranes were incubated with horseradish peroxidase-conjugated secondary antibody and the proteins detected using an enhanced chemiluminescence reagent.

### Constitution of a Risk Model

The method for constitution of a risk model was previously described (Zhang et al., 2020). Briefly, multivariate Cox proportional hazards regression analyses were performed and risk scores were constructed based on the Cox coefficients. A linear combination method was adopted to assemble the expression level and coefficient of each gene to obtain a risk score formula. The patients in the training set were stratified into a high-risk group and a low-risk group based on the median risk score.

### Univariate and Multivariate Cox Analyses

The R package “survival” was used for univariate and multivariate Cox analyses, which included *MAP3K8* expression, WHO grade, IDH status, 1p/19q codeletion, primary therapy outcome, gender, and age.

### Analysis of the Relationships Between MAP3K8, Prognosis, Diagnosis, and Clinical Phenotype

Four indicators—overall survival (OS), disease-specific survival (DSS), disease-free interval (DFI), and progression-free interval (PFI)—were selected to study the relationship between MAP3K8 expression and patient prognosis using the Kaplan–Meier method and Cox regression. Survival curves were analyzed and the plots produced by the R packages “survival” and “survminer.” The diagnostic performance of MAP3K8 was evaluated using receiver operating characteristic (ROC) curves with the R packages “pROC” and “ggplot2.”

### Function and Pathway Enrichment

The co-expressed genes with MAP3K8 from TCGA database were screened with Pearson’s correlation coefficients (|*r*| > 0.4, *p* < 0.001). Gene Ontology (GO) and Kyoto Encyclopedia of Genes and Genomes (KEGG) analyses of MAP3K8 from TCGA database were performed on co-expressed genes with the package R “clusterProfiler.” Gene set enrichment analysis (GSEA) was performed to elucidate the significant survival difference, functions, and pathways between the MAP3K8 high- and low-expression groups using the clusterProfiler package by R (v.3.6.3). |NES| > 1, *p* < 0.05, and false discovery rate (FDR) < 0.25 were considered to be statistically significant.

### Analysis of the Relationships Between MAP3K8 and Immunity

The web server TIMER (Tumor Immune Estimation Resource) was used to construct the immune cell score heatmap to represent the correlation between the immune cell score and MAP3K8 expression. Immunedeconv, an R package that integrates xCell algorithms, was utilized to make reliable immune infiltration estimations. We also used the R package “GSVA,” which integrates single-sample GSEA (ssGSEA) algorithms to estimate the abundance of immune cell types. For the correlation of MAP3K8 expression with immune inhibitors, chemokines, and chemokine receptors across human cancers, the Tumor and Immune System Interaction Database (TISIDB; http://cis.hku.hk/TISIDB) was used. To provide a comparison of the tumor infiltration levels between GBM and lower-grade glioma (LGG) with different somatic copy number alterations for MAP3K8, the somatic copy number alteration (SCNA) module was performed by the web server TIMER (https://cistrome.shinyapps.io/timer/).

### Single-Cell RNA Sequencing Data

Single-cell RNA-seq data were obtained from the Broad Institute Single-Cell Portal with study 1 (https://portals.broadinstitute.org/single_cell/study/single-cell-analysis-in- pediatric-midline-gliomas-with-histone-h3k27m-mutation), study 2 (https://singlecell.broadinstitute.org/single_cell/study/SCP393/single-cell-rna-seq-of-adult-and-pediatric-glioblastoma#study-download), and study 3 (https://singlecell.broadinstitute.org/single_cell/study/SCP50/single-cell-rna-seq-analysis-of-astrocytoma).

### Statistical Analyses

Statistical analyses were performed on GraphPad Prism 7.0 (GraphPad Software Inc., San Diego, CA, United States) and SPSS16.0 software (SPSS 16.0; SPSS Inc., Chicago, IL, United States). Statistical data acquired from TCGA were merged and conducted by R (v.3.6.3). The correlation analysis was evaluated using chi-square (*χ* ^*2*^) test, Pearson’s correlation, or Spearman’s correlation analysis. Survival analyses of data from the CGGA database were performed with the Kaplan–Meier method. The infiltration level for each SCNA category was compared with the normal using a two-sided Wilcoxon rank-sum test. Student’s *t*-test was used to determine the statistical significance of the differences between groups; the differences between more than two groups were analyzed by one-way ANOVA and the Kruskal–Wallis test. A *p*-value < 0.05 was considered statistically significant (*p < 0.05, **p < 0.01, ***p < 0.001, ****p < 0.0001).

## Results

### MAP3K8 Was Aberrantly Overexpressed in Glioma and Correlated With Poor Clinicopathologic Features of Glioma

**FIGURE 1:**
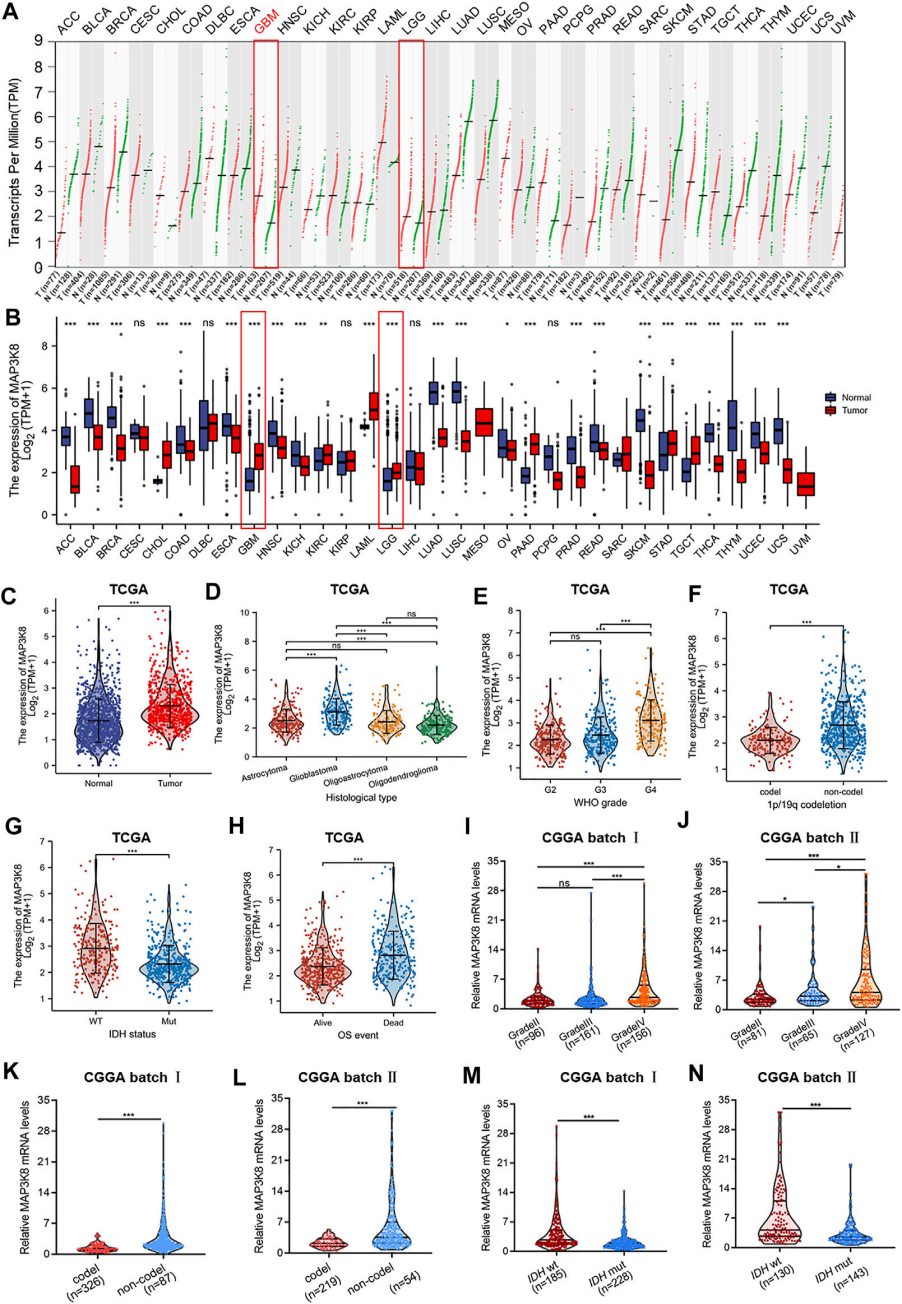
MAP3K8 was aberrantly overexpressed in glioma from The Cancer Genome Atlas (TCGA) and Chinese Glioma Genome Atlas (CGGA) databases. **(A)** Dot plot showing the gene expression profiles across all tumor samples and paired normal tissues from the Gene Expression Profiling Interactive Analysis web server. **(B)** The mRNA level of MAP3K8 analyzed across all tumor samples and paired normal tissues from TCGA database (*n* = 703) and Genotype-Tissue Expression (GTEx) database (*n* = 1,152). **(C)** The mRNA level of MAP3K8 analyzed from the CGGA database. **(D**–**H**) MAP3K8 expression in different histological types **(D)**, WHO grades (**E**), 1p/19q status **(F)**, and isocitrate dehydrogenase (*IDH*) genotypes **(G)** and the overall survival event **(H)** of glioma patients from TCGA. **(I**,**J)** MAP3K8 expression analyzed in different WHO grades from CGGA batch I **(I)** and batch II **(J)**. **(K**,**L)** MAP3K8 expression analyzed in different 1p/19q status from CGGA batch I **(K)** and batch II **(L)**. **(M**,**N)** MAP3K8 expression analyzed in different IDH genotypes from CGGA batch I **(M)** and batch II **(N)**.

The gene expression profiles across all tumor samples and paired normal tissues demonstrated that MAP3K8 was overexpressed in glioma, including GBM and LGG (Figures 1A–C). In addition, we found that the expression of MAP3K8 was obviously elevated in high-grade glioma, especially in GBM (Figures 1D, E). The *IDH* genotype, 1p/19q codeletion, and other molecular markers have been extensively used in the diagnosis and prognosis evaluation of glioma (Louis et al., 2016). We found that the messenger RNA (mRNA) level of MAP3K8 was enriched in the 1p/19q non-codeletion (Figure 1F) and *IDH* wild-type (wt) (Figure 1G) cases. Importantly, the overexpression of MAP3K8 in TCGA dataset was markedly associated with the WHO grade, *IDH* genotype, 1p/19q codeletion, and age (Table 1). In OS event, the MAP3K8 mRNA expression of the death group was higher than that of the live group (Figure 1H). In particular, the MAP3K8 expression with the WHO grade, *IDH* genotype, and 1p/19q codeletion was validated in the CGGA dataset (Figures 1I–N).

**TABLE 1:** Association between MAP3K8 mRNA expression and the clinical parameters of glioma patients from The Cancer Genome Atlas (TCGA)

	Low expression of MAP3K8	High expression of MAP3K8	*p*-value
WHO grade, *n* (%)	–	–	<0.001
G2	139 (21.9%)	85 (13.4%)	–
G3	131 (20.6%)	112 (17.6%)	–
G4	37 (5.8%)	131 (20.6%)	–
IDH status, *n* (%)	–	–	<0.001
WT	72 (10.5%)	174 (25.4%)	–
Mut	274 (39.9%)	166 (24.2%)	–
1p/19q codeletion, *n* (%)	–	–	<0.001
Codel	129 (18.7%)	42 (6.1%)	–
Non-codel	217 (31.5%)	301 (43.7%)	–
Age, median (IQR)	43 (34–56)	48.5 (35–60.25)	0.012

*WHO*, World Health Organization; *IDH*, isocitrate dehydrogenase; *WT*, wild type; *Mut*, mutant; *IQR*, interquartile range

**FIGURE 2:**
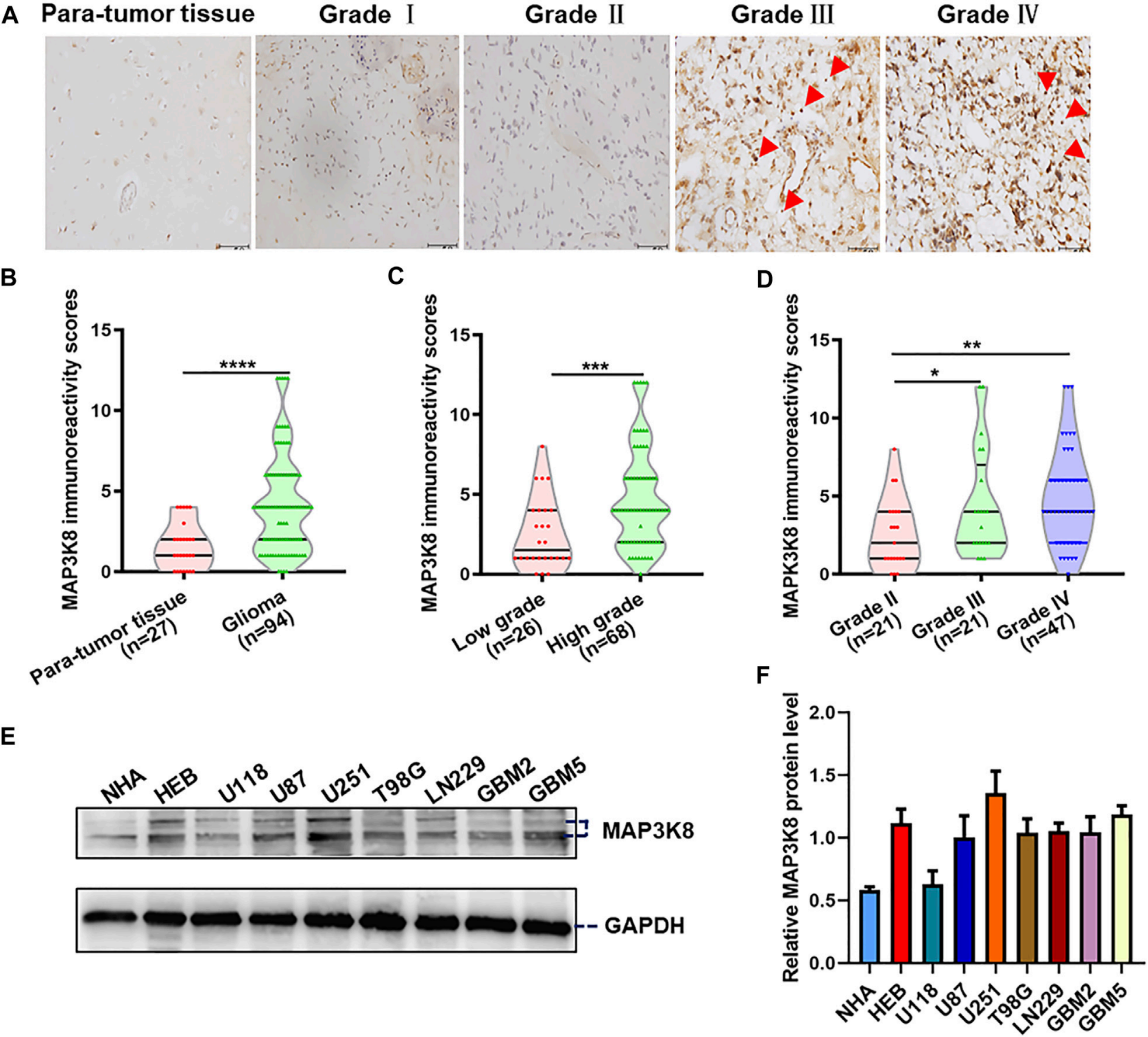
MAP3K8 was aberrantly overexpressed in glioma tissues and cell lines. **(A)** Representative immunohistochemistry (IHC) images of MAP3K8 in human para-tumor and glioma tissues. *Scale bar*, 50 μm. **(B)** MAP3K8 immunoreactivity score analyzed in human para-tumor and glioma tissues. **(C)** MAP3K8 immunoreactivity scores analyzed in low-grade and high-grade glioma tissues. **(D)** MAP3K8 immunoreactivity scores analyzed in grade II, grade III, and grade IV glioma tissues. **(E**,**F)** Expression of MAP3K8 in indicated cell lines evaluated by Western blot; statistical analysis was performed. *Error bars*, SD.

For further validation, we investigated the expression of MAP3K8 in clinical pathology specimens by IHC in 94 glioma tissues and 27 para-tumor tissues. The IHC results showed that the protein level of MAP3K8 was elevated in glioma tissues compared to para-tumor tissues (Figures 2A, B). Elevated expression of MAP3K8 was also found in high-grade glioma (Figures 2C, D). As demonstrated in Table 2, the protein level of MAP3K8 in glioma tissues was significantly associated with the WHO grade. In addition, Western blot showed that the protein level of MAP3K8 was enhanced in glioma cell lines compared with that in normal human astrocyte and glial cells (Figures 2E, F). These findings indicated that MAP3K8 was significantly overexpressed in glioma and correlated with poor clinicopathological features.

**TABLE 2:** MAP3K8 immunohistochemistry (IHC) staining and clinicopathological characteristics of 96 glioma patients

Variable	Number	MAP3K8 staining			
		Low (%)	High (%)	*χ* ^2^	*p*-value
Sex	–	–	–	0.008	0.931
Male	57	39 (68.4)	18 (31.6)	–	–
Female	37	25 (67.6)	12 (32.4)	–	–
Age (years)	–	–	–	0.154	0.130
<50	42	32 (76.2)	10 (23.8)	–	–
≥50	52	32 (61.5)	20 (38.5)	–	–
Tumor size (cm)	–	–	–	0.009	0.942
<5	30	23 (76.7)	7 (23.3)	–	–
≥5	29	22 (75.9)	7 (24.1)	–	–
WHO grade	–	–	–	4.520	**0.034**
Low (I–II)	26	22 (84.6)	4 (15.4)	–	–
High (III–IV)	68	42 (61.8)	26 (38.2)	–	–

### MAP3K8 Is an Independent Prognostic Indicator and Significantly Correlates With the Disease Progression of Glioma

**FIGURE 3:**
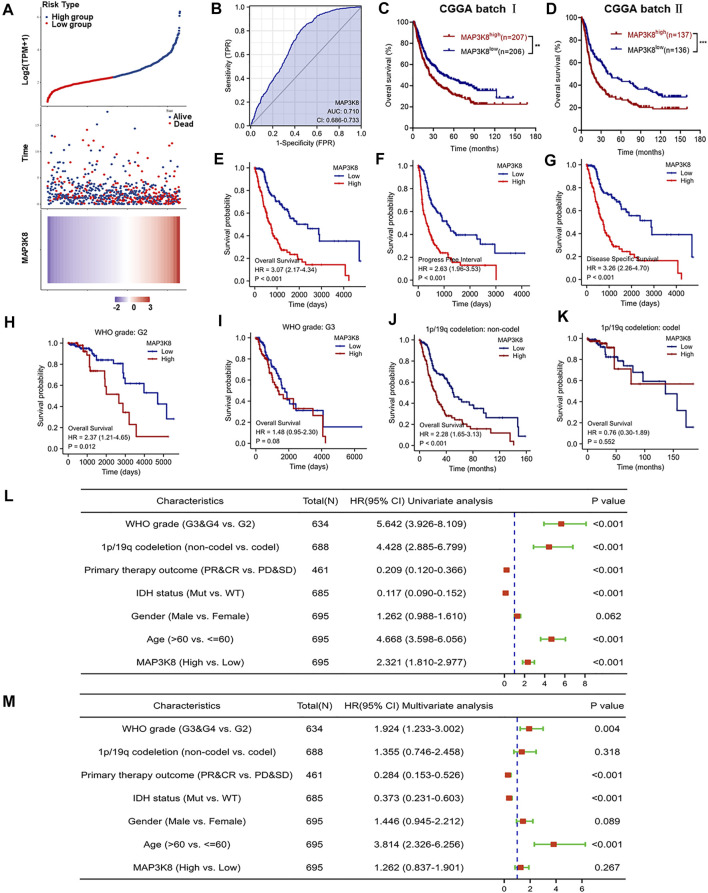
MAP3K8 is a negative prognosticator of survival in glioma. **(A)** Risk score distribution, survival overview, and heatmap of patients from The Cancer Genome Atlas (TCGA) cohorts assigned to high- and low-risk groups based on the risk scores. **(B)** Receiver operating characteristic (ROC) curve of MAP3K8 expression in the glioma cohort. **(C**–**E)** Overall survival analysis in datasets from the Chinese Glioma Genome Atlas (CGGA) batch I **(C)** and batch II **(D)** and TCGA **(E)**. **(F**,**G)** Disease-specific survival (DSS) **(F)** and progression-free interval (PFI) **(G)** in TCGA dataset. **(H**,**I)** Survival analysis of MAP3K8 mRNA expression in different WHO grades from TCGA dataset. **(J**,**K)** Survival analysis of MAP3K8 mRNA expression in different 1p/19q codeletion status from TCGA dataset. **(L**,**M)** Forest plot showing the univariate **(L)** and multivariate **(M)** Cox regression models of prognosis for MAP3K8 in TCGA cohort.

To further explore the role of MAP3K8 in prognosis, glioma patients were divided into high- and low-risk groups according to the cutoff value of the median risk score. Poorer prognosis and more deaths occurred in the high-risk group than in the low-risk group (Figure 3A). ROC curve analysis of MAP3K8 demonstrated that the area under the ROC curve (AUC) was 0.710, which indicated high diagnostic value of MAP3K8 expression in glioma (Figure 3B). Analysis of OS in the CGGA (Figures 3C, D) and TCGA (Figure 3E) datasets showed that MAP3K8 was negatively correlated with the prognosis of glioma. The expression MAP3K8 was also significantly associated with the PFI and DSS of glioma patients in TCGA dataset (Figures 3F, G). Moreover, patients with elevated MAP3K8 expressions in grade 2 and 1p/19q non-codeletion group had poorer prognosis (Figures 3H–K). Univariate Cox regression analysis demonstrated that MAP3K8 expression, WHO grade, 1p/19q codeletion, and age were correlated with the OS of glioma patients (Figure 3L). Multivariate Cox regression analysis demonstrated that WHO grade and age were correlated with the OS of glioma patients (Figure 3M).The results strongly indicated that MAP3K8 was an independent prognostic factor and correlated with the disease progression of glioma.

### Function and Pathway Enrichment Analysis of MAP3K8 in Glioma

**FIGURE 4:**
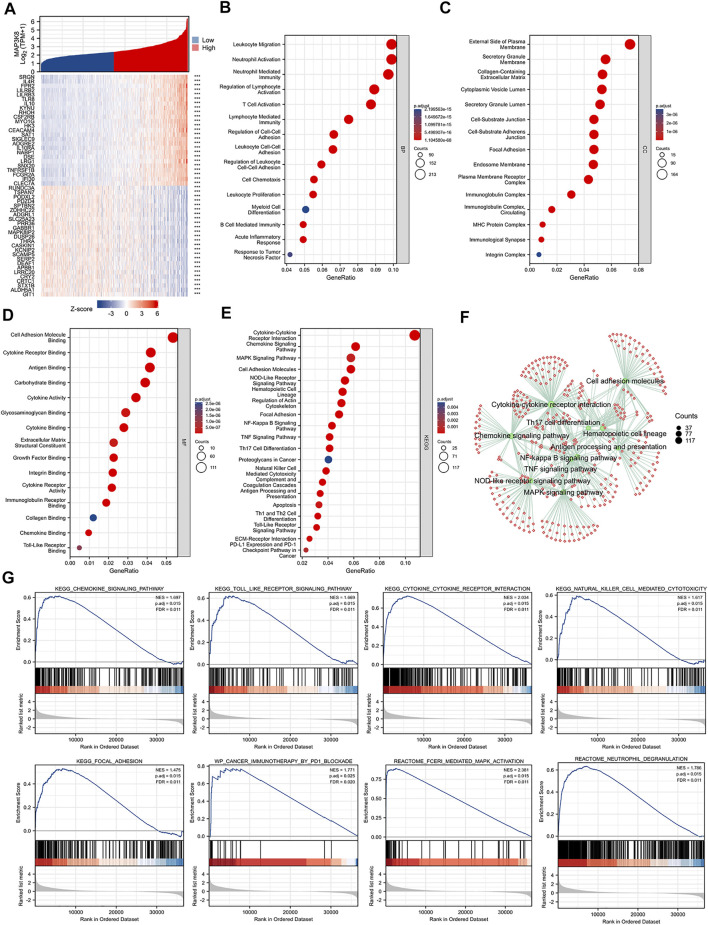
Gene Ontology (GO), Kyoto Encyclopedia of Genes and Genomes (KEGG), and gene set enrichment analysis (GSEA) of MAP3K8 in TCGA dataset. **(A)** Top 25 genes most positively or negatively associated with MAP3K8 shown in a heatmap. **(B**–**D)** GO enrichment analysis for biological process **(B)**, cellular component **(C)**, and molecular function **(D)** of MAP3K8 and its co-expression genes. **(E**,**F)** KEGG pathway enrichment analysis of MAP3K8 and its co-expression genes. **(G)** GSEA of MAP3K8 and its co-expression genes.

Single-gene correlation analysis showed the top 25 genes positively and negatively associated with MAP3K8 (Figure 4A). GO enrichment, KEGG pathway analysis, and GSEA were performed to explore the biological functions of MAP3K8 in glioma. As shown in Figure 4B, MAP3K8 co-expressed genes were mainly enriched in immune-related biological processes such as neutrophil activation, leukocyte migration, neutrophil-mediated immunity, T-cell activation, leukocyte cell–cell adhesion, B-cell-mediated immunity, myeloid cell differentiation, and regulation of cell–cell adhesion. For cellular component, the immunoglobulin complex, plasma membrane receptor complex, MHC protein complex, focal adhesion, and integrin complex were enriched (Figure 4C).MAP3K8 co-expressed genes were mainly enriched in immune-related molecular functions such as antigen binding, cytokine binding, cytokine receptor binding, immunoglobulin receptor binding, integrin binding, chemokine binding, and Toll-like receptor binding (Figure 4D). Analysis of KEGG pathway enrichment showed that MAP3K8 co-expressed genes were highly enriched in the MAPK signaling pathway, NF-kappa B signaling pathway, Toll-like receptor signaling pathway, cytokine–cytokine receptor interaction, chemokine signaling pathway, T helper 2 (Th2) cell differentiation, cell adhesion molecules, programmed death-ligand 1 (PD-L1) expression, and programmed death-1 (PD-1) checkpoint pathway in cancer (Figures 4E, F). In addition, GSEA also showed that neutrophil degranulation, the chemokine signaling pathway, Toll-like receptor cascades, FceRI-mediated MAPK activation, and cancer immunotherapy by PD1_blockade were significantly enriched (Figure 4G). These results suggested that MAP3K8 might participate in the tumor immune microenvironment and immune regulation.

### MAP3K8 Expression Is Mainly Enriched in Microglia/Macrophage Cells From Single-Cell RNA-Sequencing Data

**FIGURE 5:**
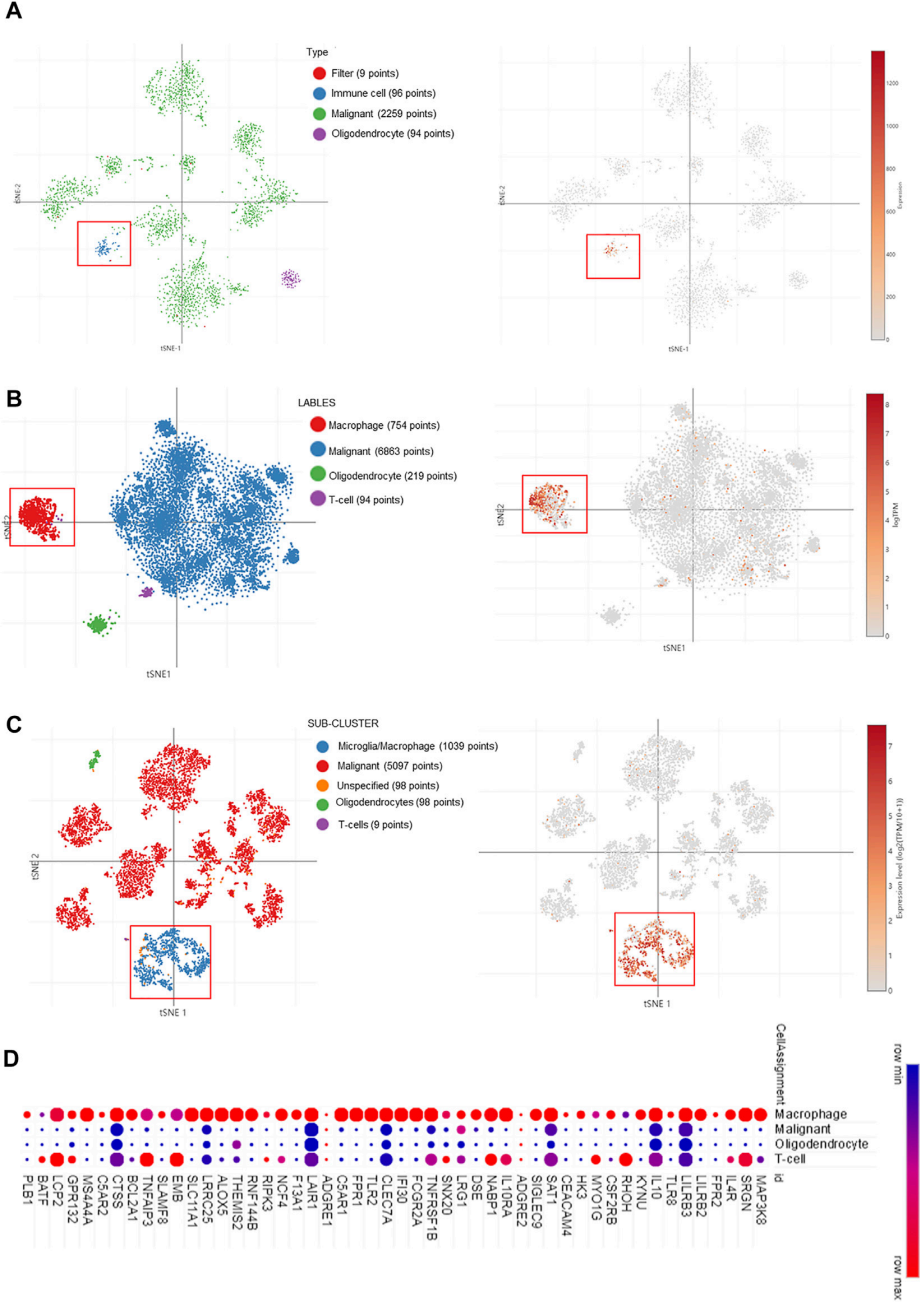
MAP3K8 expression in glioma from single-cell RNA sequencing data. **(A**–**C)** MAP3K8 expression in different sub-clusters of glioma from study 1 **(A)**, study 2 **(B)**, and study 3 **(C)**. **(D)** The top 25 genes positively associated with MAP3K8 mainly enriched in different sub-clusters of glioma.

Taking into account the heterogeneity of the tumor, we explored the expression of MAP3K8 from the single-cell RNA-seq data. As shown in Figure 5A, MAP3K8 was enriched in immune cells from single-cell analysis in pediatric midline gliomas with histone H3K27M mutation 4058 cells. In two other single-cell RNA-seq data, MAP3K8 was enriched in both malignant and macrophage cells (Figures 5B, C). In addition, the top 25 genes positively associated with MAP3K8 were mainly enriched in macrophage cells (Figure 5D). The IHC results also showed that MAP3K8 was expressed in malignant and immune cells (Figure 2E).

### Correlation Analysis of MAP3K8 With Immune Infiltration in Glioma

**FIGURE 6:**
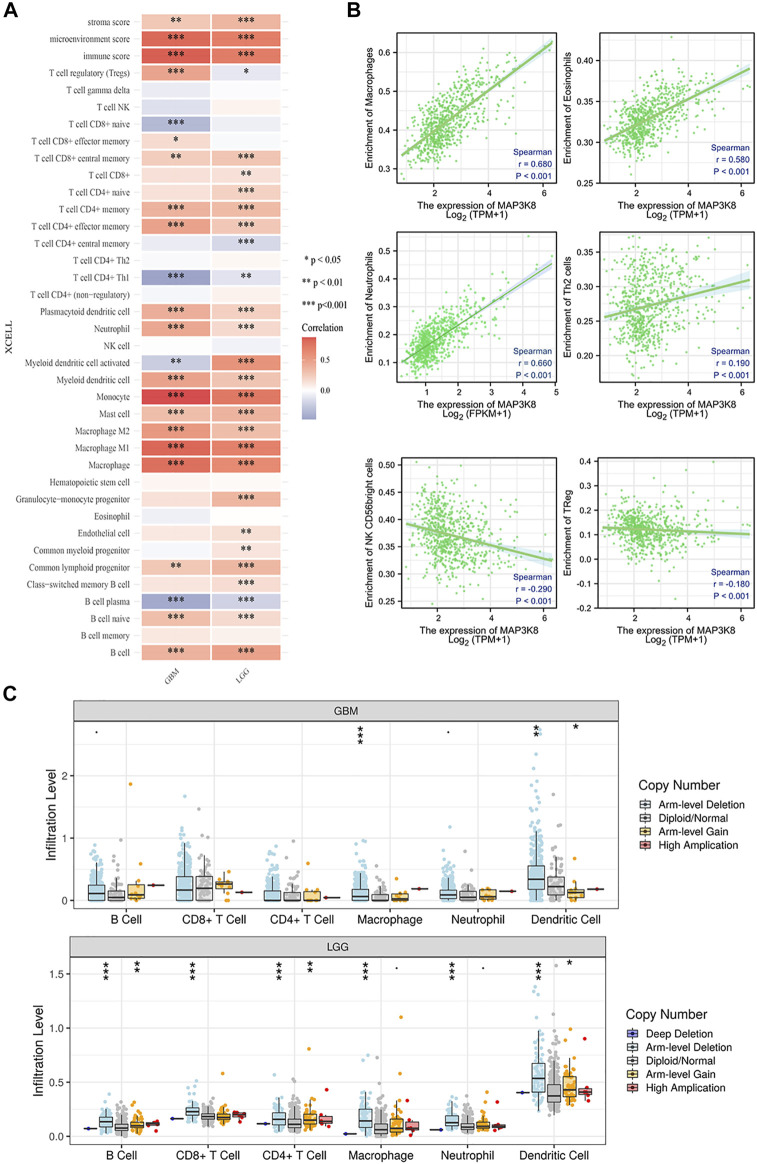
Correlation analysis of MAP3K8 with immune infiltration in glioma. **(A)** Heatmap of the immune cell scores, where *different colors* represent the correlation between the immune cell scores and MAP3K8 expression. The xCell algorithms were utilized to make reliable immune infiltration estimations. **(B)** Analysis of the relationship between MAP3K8 expression and immune infiltration in glioma. The immune cell infiltration level was quantified by ssGSEA algorithms. **(C)** Somatic copy number alteration (SCNA) module showing the comparison of the tumor infiltration levels among tumors with different somatic copy number alterations for MAP3K8. SCNAs were defined using GISTIC 2.0.

We firstly analyzed the relationship between MAP3K8 expression and immune infiltration in glioma. As shown in Figure 6A, the expression of MAP3K8 was positively correlated with the enrichment of memory CD4^+^ T cells, effector memory CD4^+^ T cells, plasmacytoid dendritic cells, neutrophils, myeloid dendritic cells, mast cells, macrophages, M2 macrophage, M1 macrophage, and B cells in both GBM and LGG. In total glioma, the expression of MAP3K8 was positively correlated with the enrichment of macrophages, eosinophils, neutrophils, and Th2 cells. The expression of MAP3K8 was negatively correlated with the enrichment of natural killer (NK) CD56 bright cells and regulatory T cells (Figure 6B). We also performed a comparison of the immune infiltration levels between GBM and LGG with different SCNAs for MAP3K8. The infiltration levels of macrophages and dendritic cells with arm-level deletion were significantly enriched compared with the normal in both GBM and LGG (Figure 6C).

### Correlations Between the Expression of MAP3K8 and Immune Checkpoint Molecules, Chemokines, and Chemokine Receptors

**FIGURE 7:**
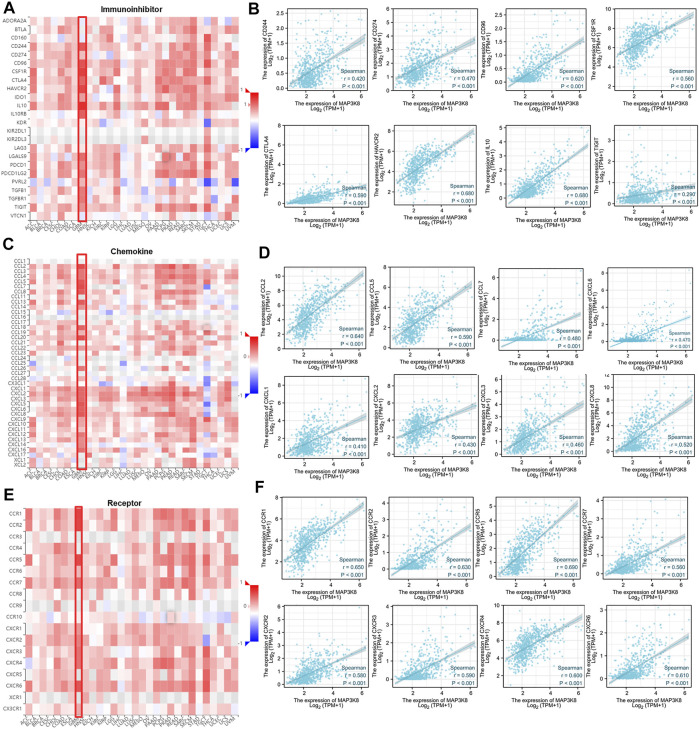
Correlations between MAP3K8 expression and the expressions of immune inhibitors, chemokines, and chemokine receptor. **(A**,**C**,**E)** Correlations between the expressions of MAP3K8 and immune inhibitors **(A)**, chemokines **(C)**, and chemokine receptors **(E)** across human cancers. **(B**,**D**,**F)** Correlations between the expressions of MAP3K8 and immune inhibitors **(B)**, chemokines **(D)**, and chemokine receptors **(F)** in glioma from The Cancer Genome Atlas (TCGA) dataset.

**FIGURE 8:**
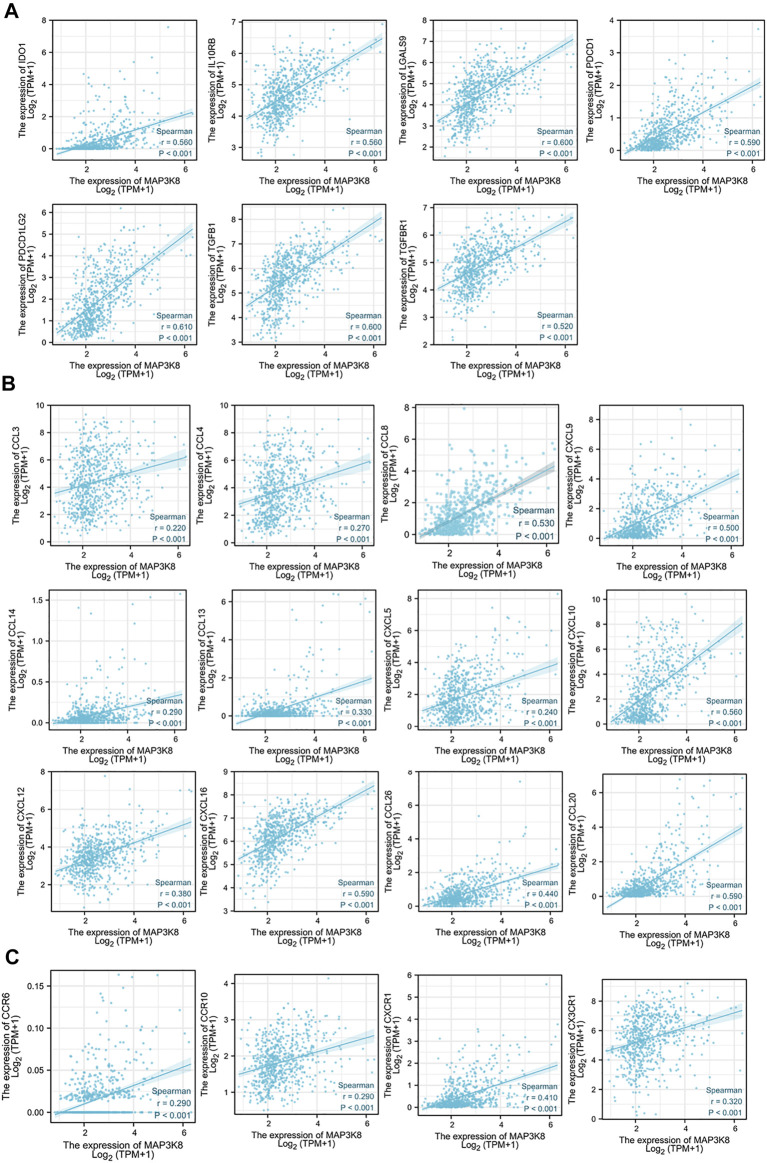
Correlations of MAP3K8 expression with the expressions of immune inhibitors, chemokines, and chemokine receptors in The Cancer Genome Atlas (TCGA) dataset. **(A**–**C)** Correlations between the expressions of MAP3K8 and immune inhibitors **(A)**, chemokines **(B)** and chemokine receptors **(C)** in glioma from TCGA dataset. The correlation analysis was evaluated by Spearman’s correlation.

To further explore the role of MAP3K8 in tumor immune response, the correlations between the expression of MAP3K8 and immunomodulators were calculated. The web server TIMER was used to assess the correlation between MAP3K8 expression and immune checkpoint molecules, chemokines, and chemokine receptors (Figures 7A, C, E). As shown in Figures 7A, B, 8A, the expression of MAP3K8 was positively correlated with the expression of the main immune checkpoint inhibitor molecules in GBM such as CD244 (*r* = 0.42, *p* < 0.001), CD274 (*r* = 0.47, *p* < 0.001), CD96 (*r* = 0.62, *p* < 0.001), CSF1R (*r* = 0.56, *p* < 0.001), CTLA4 (*r* = 0.59, *p* < 0.001), HAVCR2 (*r* = 0.68, *p* < 0.001), IDO1 (*r* = 0.56, *p* < 0.001), IL10 (*r* = 0.68, *p* < 0.001), IL10RB (*r* = 0.56, *p* < 0.001), LGSLS9 (*r* = 0.60, *p* < 0.001), PDCD1 (*r* = 0.59, *p* < 0.001), PDCD1LG2 (*r* = 0.61, *p* < 0.001), TGFB1 (*r* = 0.60, *p* < 0.001), TGFBR1 (*r* = 0.52, *p* < 0.001), and TIGIT (*r* = 0.29, *r* = 0.001).

We also analyzed the correlations between MAP3K8 expression and chemokines and receptors. As shown in Figures 7C, D, 8B, the expression of MAP3K8 was positively correlated with the main chemokines such as CCL2, CCL3, CCL4, CCL5, CCL7, CCL8, CCL13, CCL14, CCL20, CCL26, CXCL1, CXCL2, CXCL3, CXCL5, CXCL6, CXCL8, CXCL9, CXCL10, CXCL12, CXCL13, and CXCL16. In addition, the expression of MAP3K8 was also positively correlated with the main chemokine receptors such as CCR1, CCR2, CCR5, CCR6, CCR7, CCR10, CXCR1, CXCR2, CXCR3, CXCR4, CXCR6, and CX3CR1 (Figures 7E–F, 8C). Our results indicated that MAP3K8 might play an essential role in tumor immunoregulation and work as a putative drug target in glioma.

## Discussion

It has been reported that MAP3K8 was overexpressed in several types of cancer, including **s**kin cancer, prostate cancer, breast cancer, squamous cell carcinoma, ovarian cancer, hepatocellular carcinoma, colorectal cancer, endometrial cancer, gastric cancer, nasopharyngeal carcinomas, anaplastic large-cell lymphoma, colitis-associated cancer, bladder cancer, and cervical cancer (Sourvinos et al., 1999; Jeong et al., 2011; Gruosso et al., 2015; Lee et al., 2015; Li et al., 2015; Pyo et al., 2018). To date, the expression, clinical significance, biological role, and underlying molecular mechanisms of MAP3K8 in glioma have not been investigated yet. Our study demonstrated that MAP3K8 was also overexpressed in GBM and LGG. In clinical pathology specimens, the protein level of MAP3K8 was elevated in glioma tissues, in contrast to para-tumor tissues. Elevated expression of MAP3K8 was also found in high-grade glioma and was significantly associated with the WHO grade. The abundance of MAP3K8 expression may be efficient for biomarker identification. We found that MAP3K8 was mainly enriched in the microglia/macrophage cells from the single-cell RNA-seq data. The top 25 genes positively associated with MAP3K8 were also mainly enriched in macrophage cells. Our results indicated that the role of MAP3K8 in macrophages might be a promising research direction in glioma.

Efficient prognostic biomarkers in patients provide important information regarding cancer aggressiveness or the clinical outcomes and influence the management and treatment decisions for a specific glioma patient (Jelski and Mroczko, 2021; Rzhevskiy et al., 2021). Currently, there is still lack of accurate and efficient diagnostic and prognostic biomarkers for glioma. Our results revealed that MAP3K8 was an independent prognostic indicator and significantly correlated with the disease progression and poor clinicopathological features of glioma. The expression and the prognostic implication of MAP3K8 were examined using online public databases (TCGA and CGGA) and validated in our clinical pathology specimens. MAP3K8 will be a promising diagnostic and prognostic biomarker that allows the natural course of a tumor to be predicted, distinguishing “good outcome” tumors from “poor outcome” tumors.

The function and pathway enrichment analyses of MAP3K8 in glioma indicated that MAP3K8 might participate in the tumor immune microenvironment and immune regulation. MAP3K8 co-expressed genes were mainly enriched in immune-related biological processes, including neutrophil activation, leukocyte migration, neutrophil-mediated immunity, lymphocyte-mediated immunity, T-cell activation, leukocyte cell–cell adhesion, regulation of leukocyte cell–cell adhesion, B-cell-mediated immunity, and myeloid cell differentiation. MAP3K8 has been reported to regulate multiple neutrophil antimicrobial pathways, including inflammatory cytokine secretion and oxidative burst (Acuff et al., 2017). More works are needed to explore the role of MAP3K8 in tumor-infiltrating neutrophil-mediated tumor immune response in glioma. Although MAP3K8 is considered to play a critical role in inflammation response and immune diseases, the importance of MAP3K8-driven inflammation in tumorigenesis and tumor immunity, as well as the underlying mechanisms that drive these processes, is not fully understood (Njunge et al., 2020). The role of MAP3K8 in regulating neutrophil activation, leukocyte migration, leukocyte cell–cell adhesion, and B-cell-mediated immunity for glioma immunoregulation remains unclear.

As is well known, tumor immune cell infiltration could influence the efficacy of chemotherapy, radiotherapy, or immunotherapy and the prognosis of cancer patients (Fridman et al., 2012; Gajewski et al., 2013; Domingues et al., 2016). The expression of MAP3K8 was positively correlated with the enrichment of memory CD4^+^ T cells, effector memory CD4^+^ T cells, plasmacytoid dendritic cells, neutrophils, myeloid dendritic cells, mast cells, macrophages, M2 macrophage, and M1 macrophage in glioma. These results indicated that MAP3K8 might partially participate in the tumor immune infiltration of glioma. Meanwhile, MAP3K8 and the top 25 genes positively associated with MAP3K8 were also mainly enriched in macrophage cells. In multiple myeloma, MAP3K8 activation promoted the acquisition of the M2 macrophage phenotype that promotes immunosuppression and inflammation (Hope et al., 2014; Jensen et al., 2015). In glioma, MAP3K8 might act as an essential modulator of polarization and function for tumor-associated macrophages.

Immune checkpoint inhibitory molecules are known to mediate immune evasion and maintain the malignant behaviors of cancer cells, including self-renewal, epithelial–mesenchymal transition, metastasis, drug and radiotherapy resistance, anti-apoptosis, and angiogenesis (Pardoll, 2012; Wei et al., 2018; Zhang and Zheng, 2020). In the tumor microenvironment, different immune cell subsets are recruited *via* the interactions between chemokines and chemokine receptors to directly or indirectly affect tumor immunity, therapy, and patient outcomes and participate in tumor progression (Pardoll, 2012). At present, immune checkpoint molecules, chemokines, and chemokine receptors are used as new targets for cancer immunotherapy (Pardoll, 2012; Mollica Poeta et al., 2019). The expression of MAP3K8 was positively correlated with the expression of the main immune checkpoint inhibitory molecules, chemokines, and chemokine receptors. These data indicated that MAP3K8 might function as a biomarker with the potential to be an integrative or specific target of cancer immunotherapy in glioma. In view of the correlation analysis of MAP3K8 with immune infiltration, the expressions of immune checkpoint molecules, chemokines, and chemokine receptors in glioma, MAP3K8 might play an essential role in tumor immunity, and the inhibition of MPA3K8 is a plausible strategy for glioma immunotherapy.

## Conclusion

Collectively, our study is the first to provide evidence that MAP3K8 was aberrantly overexpressed in glioma and correlated with poor clinicopathological features. MAP3K8 might serve as a valuable diagnostic and prognostic indicator. In view of the comprehensive regulatory role of MAP3K8 in tumor immunity, therapeutic regimen blocking MAP3K8 might be a promising strategy for the development of more integrative and specific cancer immunotherapy for glioma.

## Data Availability

The raw data supporting the conclusion of this article will be made available by the authors, without undue reservation.
