# Supplementary material for: MAP3K8 Is a Prognostic Biomarker and Correlated With Immune Response in Glioma
Source: Front Mol Biosci. 2021 Dec 23;8:779290. doi: 10.3389/fmolb.2021.779290 (PMC8733582; doi:10.3389/fmolb.2021.779290)
Supplement: Supplementary file 1 [file DataSheet1.DOCX]

**MAP3K8 is a prognostic biomarker and correlated with immune response in glioma**

Supplementary **Fig.1**

**
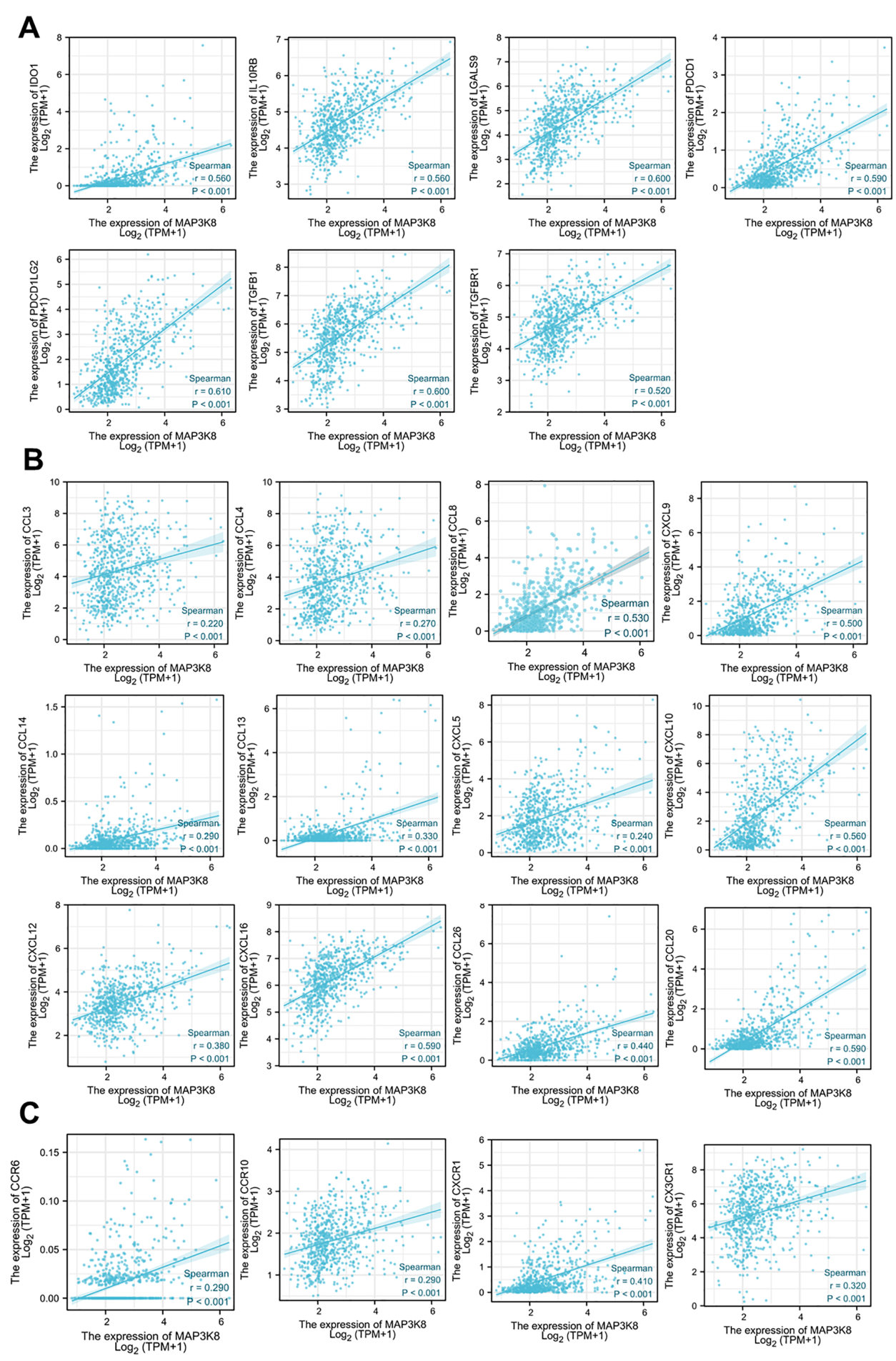
**

1. **Correlations MAP3K8 expression and the expression of immunoinhibitor, chemokine and chemokine receptor.** (A) The relationship between MAP3K8 expression and immunoinhibitors in glioma from TCGA data. (B) The relationship between MAP3K8 expression and chemokine in glioma from TCGA data. (C) The relationship between MAP3K8 expression and chemokine receptor in glioma from TCGA data.
